# Supplementary material for: Bushfires and Mothers’ Mental Health in Pregnancy and Recent Post-Partum
Source: Int J Environ Res Public Health. 2023 Dec 20;21(1):7. doi: 10.3390/ijerph21010007 (PMC10815782; doi:10.3390/ijerph21010007)
Supplement: Supplementary file 1 [file ijerph-21-00007-s001.zip › ijerph-2623375-supplementary.pdf]

SUPPLEMENTARY MATERIALS

Table S1. Fully adjusted models testing association between bushfire and smoke exposure, mental health outcomes.

	Dependent variable:							
	WHO-5		Depression		Anxiety		Stress	
Intercept	67.024***	67.289***	3.164**	3.080**	5.701***	5.563***	3.941**	3.767**
	p = 0.000	p = 0.000	p = 0.004	p = 0.005	p = 0.000	p = 0.000	p = 0.003	p = 0.005
Age, yrs	-0.209	-0.216	-0.069**	-0.067*	-0.123***	-0.120***	-0.075*	-0.071*
	p = 0.203	p = 0.187	p = 0.011	p = 0.014	p = 0.00000	p = 0.00000	p = 0.021	p = 0.028
Parity, number	-1.479	-1.409	0.425**	0.408**	0.508***	0.492***	0.599***	0.570***
	p = 0.072	p = 0.088	p = 0.002	p = 0.003	p = 0.00001	p = 0.00001	p = 0.0003	p = 0.0005
Tertiary education (yes)	-0.710	-0.784	-0.293	-0.274	-0.443*	-0.420	-0.295	-0.260
	p = 0.667	p = 0.635	p = 0.280	p = 0.313	p = 0.042	p = 0.054	p = 0.365	p = 0.425
Income (high)	0.057	-0.160	-0.070	-0.020	-0.632	-0.588	1.092	1.176
	p = 0.988	p = 0.965	p = 0.905	p = 0.973	p = 0.178	p = 0.209	p = 0.120	p = 0.094
Ever smoker (no)	0.157	0.069	-0.076	-0.057	-0.339	-0.326	-0.281	-0.251
	p = 0.924	p = 0.967	p = 0.778	p = 0.833	p = 0.117	p = 0.132	p = 0.385	p = 0.437
Ever smoker (unknown)	0.516	0.533	0.117	0.111	0.450	0.442	0.335	0.325
	p = 0.808	p = 0.801	p = 0.737	p = 0.748	p = 0.107	p = 0.113	p = 0.422	p = 0.436
Hypertension (yes)	4.584	4.793	-0.190	-0.236	0.806	0.770	-0.384	-0.460
	p = 0.275	p = 0.253	p = 0.783	p = 0.732	p = 0.146	p = 0.164	p = 0.642	p = 0.578
Prior anxiety (yes)	-11.209***	-11.230***	1.080***	1.085***	1.331***	1.337***	2.428***	2.437***
	p = 0.000	p = 0.000	p = 0.00002	p = 0.00002	p = 0.000	p = 0.000	p = 0.000	p = 0.000
Prior anxiety (unknown)	2.501	2.063	-0.807	-0.725	0.202	0.220	-0.494	-0.386
	p = 0.634	p = 0.694	p = 0.349	p = 0.400	p = 0.772	p = 0.750	p = 0.634	p = 0.709
Prior depression (yes)	-5.540**	-5.551**	1.802***	1.804***	0.755**	0.756**	1.270***	1.273***
	p = 0.004	p = 0.004	p = 0.000	p = 0.000	p = 0.003	p = 0.003	p = 0.001	p = 0.001
Pregnancy phase (pregnant)	0.191	0.273	0.885*	0.866*	-0.113	-0.129	0.460	0.429
	p = 0.940	p = 0.914	p = 0.032	p = 0.036	p = 0.732	p = 0.696	p = 0.354	p = 0.386
Pregnancy phase (new baby)	-1.457	-1.280	1.292**	1.253**	0.190	0.162	1.056	0.995
	p = 0.594	p = 0.640	p = 0.005	p = 0.006	p = 0.599	p = 0.653	p = 0.051	p = 0.065
Bushfire exposure (acute)	-2.032**		0.413***		0.206**		0.603***	
	p = 0.002		p = 0.0001		p = 0.013		p = 0.00001	
Bushfire exposure (broad)		-1.014**		0.214***		0.131**		0.325***
		p = 0.002		p = 0.00004		p = 0.002		p = 0.00000

Smoke exposure (moderate)	-1.474	-1.443	-0.189	-0.199	0.405	0.386	0.722	0.699
	p = 0.590	p = 0.598	p = 0.674	p = 0.657	p = 0.262	p = 0.284	p = 0.181	p = 0.195
Smoke exposure (severe)	-2.346	-2.223	-0.082	-0.115	0.465	0.421	0.783	0.719
	p = 0.369	p = 0.396	p = 0.849	p = 0.789	p = 0.178	p = 0.222	p = 0.129	p = 0.163
Observations	919	919	919	919	919	919	919	919
Log Likelihood	-4,011.889	-4,011.949	-2,351.101	-2,350.576	-2,151.008	-2,149.090	-2,520.680	-2,518.779
Akaike Inf. Crit.	8,055.778	8,055.898	4,734.202	4,733.151	4,334.016	4,330.180	5,073.359	5,069.558
Note:						*p<0.05;* ***p<0.0125;*** p<0.001		

Table S2. Associations between bushfire/smoke and COVID-19 exposures, and mental health outcomes.

	Dependent variable:							
	WHO-5		Depression		Anxiety		Stress	
Intercept	57.653***	58.321***	4.963***	4.767***	6.397***	6.185***	7.188***	6.932***
	p = 0.000	p = 0.000	p = 0.00001	p = 0.00001	p = 0.000	p = 0.000	p = 0.00000	p = 0.00000
Age, yrs	-0.060	-0.062	-0.088**	-0.087**	-0.147***	-0.144***	-0.101**	-0.099**
	p = 0.740	p = 0.733	p = 0.003	p = 0.004	p = 0.000	p = 0.000	p = 0.005	p = 0.006
Parity, number	-1.716	-1.634	0.375*	0.349*	0.462***	0.436***	0.538**	0.501**
	p = 0.067	p = 0.081	p = 0.014	p = 0.022	p = 0.0002	p = 0.0003	p = 0.004	p = 0.007
Tertiary education (yes)	1.963	2.105	-0.640*	-0.658*	-0.938***	-0.946***	-0.937**	-0.948**
	p = 0.272	p = 0.240	p = 0.028	p = 0.023	p = 0.00005	p = 0.00005	p = 0.009	p = 0.008
Pregnancy phase (pregnant)	1.603	1.482	0.394	0.413	-0.157	-0.149	0.055	0.067
	p = 0.557	p = 0.586	p = 0.372	p = 0.347	p = 0.654	p = 0.669	p = 0.919	p = 0.901
Pregnancy phase (new baby)	0.435	0.461	0.563	0.542	-0.005	-0.027	0.536	0.494
	p = 0.884	p = 0.877	p = 0.243	p = 0.259	p = 0.991	p = 0.943	p = 0.363	p = 0.399
Bushfire exposure (acute)	-1.147		0.395***		0.159		0.546***	
	p = 0.106		p = 0.001		p = 0.082		p = 0.0002	
Bushfire exposure (broad)		-0.633		0.224***		0.126**		0.317***
		p = 0.079		p = 0.0002		p = 0.007		p = 0.00001
Smoke exposure (moderate)	-4.788	-4.405	-0.065	-0.165	0.695	0.614	1.169	1.037
	p = 0.132	p = 0.166	p = 0.900	p = 0.748	p = 0.090	p = 0.132	p = 0.064	p = 0.098
Smoke exposure (severe)	-7.153*	-6.626*	0.139	-0.003	0.897*	0.777*	1.408*	1.222*
	p = 0.019	p = 0.030	p = 0.778	p = 0.995	p = 0.022	p = 0.047	p = 0.020	p = 0.042
Covid exposure (acute)	-0.574		0.098		0.151		0.145	
	p = 0.706		p = 0.691		p = 0.439		p = 0.630	
Covid exposure (broad)		-1.048		0.236*		0.181*		0.280*
		p = 0.102		p = 0.023		p = 0.028		p = 0.027
Observations	777	777	777	777	777	777	777	777
Log Likelihood	-3,422.794	-3,421.168	-2,008.626	-2,004.324	-1,829.559	-1,825.006	-2,164.553	-2,159.537
Akaike Inf. Crit.	6,865.588	6,862.336	4,037.252	4,028.648	3,679.119	3,670.013	4,349.107	4,339.074
Note:								*p<0.05;* ***p<0.0125;*** p<0.001

Table S3. Interactions between acute bushfire exposure and COVID-19 exposure.

	Dependent variable:							
	WHO-5		Depression		Anxiety		Stress	
Intercept	53.431***	54.249***	5.319***	5.103***	6.846***	6.675***	8.445***	8.190***
	p = 0.000	p = 0.000	p = 0.000	p = 0.00000	p = 0.000	p = 0.000	p = 0.000	p = 0.000
Age, yrs	-0.069	-0.085	-0.088**	-0.084**	-0.144***	-0.141***	-0.098**	-0.092**
	p = 0.701	p = 0.636	p = 0.003	p = 0.004	p = 0.000	p = 0.000	p = 0.007	p = 0.010
Parity, number	-1.659	-1.593	0.365*	0.344*	0.446***	0.429***	0.522**	0.496**
	p = 0.077	p = 0.090	p = 0.016	p = 0.023	p = 0.0003	p = 0.0004	p = 0.005	p = 0.008
Tertiary education (yes)	2.373	2.275	-0.698*	-0.660*	-1.007***	-0.971***	-1.054**	-0.998**
	p = 0.184	p = 0.204	p = 0.016	p = 0.023	p = 0.00002	p = 0.00003	p = 0.003	p = 0.005
Covid exposure (broad)	-0.360	-0.579	0.137	0.183	0.183	0.158	0.080	0.068
	p = 0.708	p = 0.546	p = 0.376	p = 0.236	p = 0.140	p = 0.199	p = 0.675	p = 0.718
Bushfire exposure (acute)	-0.536		0.303		0.160		0.353	
	p = 0.581		p = 0.054		p = 0.200		p = 0.066	
Covid x acute bushfire	-0.838		0.108		0.026		0.233	
	p = 0.200		p = 0.307		p = 0.760		p = 0.072	
Bushfire exposure (broad)		-0.438		0.205**		0.117		0.220*
		p = 0.388		p = 0.013		p = 0.071		p = 0.028
Covid x broad bushfire		-0.287		0.023		0.018		0.107
		p = 0.376		p = 0.656		p = 0.656		p = 0.094
Observations	777	777	777	777	777	777	777	777
Log Likelihood	-3,423.924	-3,424.072	-2,006.087	-2,005.100	-1,829.776	-1,827.290	-2,163.441	-2,161.147
Akaike Inf. Crit.	6,861.847	6,862.143	4,026.175	4,024.200	3,673.553	3,668.579	4,340.883	4,336.295
Note:								*p<0.05;* ***p<0.0125;*** p<0.001

Table S4. Interactions between bushfire exposure and pregnancy stage (1st, 2nd or 3rd semester) in predicting mental health outcomes.

	Dependent variable:							
	WHO-5		Depression		Anxiety		Stress	
Intercept	55.964***	55.889***	5.130***	5.119***	6.148***	6.103***	7.895***	7.853***
	p = 0.000	p = 0.000	p = 0.00001	p = 0.00001	p = 0.000	p = 0.000	p = 0.00000	p = 0.00000
Age, yrs	-0.043	-0.048	-0.082**	-0.079*	-0.133***	-0.130***	-0.115**	-0.110**
	p = 0.833	p = 0.813	p = 0.012	p = 0.016	p = 0.00000	p = 0.00000	p = 0.005	p = 0.007
Parity, number	-1.466	-1.419	0.377*	0.358*	0.372**	0.356**	0.531**	0.500*
	p = 0.168	p = 0.184	p = 0.028	p = 0.038	p = 0.007	p = 0.010	p = 0.013	p = 0.019
Tertiary education (yes)	1.977	2.006	-0.593	-0.585	-0.753**	-0.723**	-0.962*	-0.923*
	p = 0.313	p = 0.307	p = 0.061	p = 0.065	p = 0.003	p = 0.005	p = 0.014	p = 0.019
Trimester (second)	-2.299	-1.920	0.761	0.614	-0.008	-0.098	1.309*	1.173*
	p = 0.449	p = 0.523	p = 0.120	p = 0.204	p = 0.985	p = 0.800	p = 0.031	p = 0.050
Trimester (third)	-1.068	-1.418	0.578	0.629	0.049	-0.082	1.050	0.945
	p = 0.723	p = 0.634	p = 0.233	p = 0.190	p = 0.900	p = 0.829	p = 0.081	p = 0.112
Bushfire exposure (acute)	-0.542		0.383		0.447*		0.924**	
	p = 0.742		p = 0.149		p = 0.035		p = 0.005	
Bushfire exposure (broad)		-0.197		0.177		0.216		0.461**
		p = 0.821		p = 0.206		p = 0.053		p = 0.008
Smoke exposure (moderate)	-1.563	-1.603	-0.629	-0.635	0.318	0.300	0.240	0.187
	p = 0.645	p = 0.638	p = 0.251	p = 0.247	p = 0.465	p = 0.492	p = 0.723	p = 0.783
Smoke exposure (severe)	-2.071	-2.019	-0.471	-0.510	0.373	0.329	0.222	0.127
	p = 0.524	p = 0.537	p = 0.369	p = 0.333	p = 0.372	p = 0.432	p = 0.733	p = 0.845
Acute exposure in 2nd trimester	-0.441		-0.123		-0.256		-0.708	
	p = 0.825		p = 0.701		p = 0.317		p = 0.075	
Acute exposure in 3rd trimester	-1.977		0.290		-0.269		-0.299	
	p = 0.324		p = 0.369		p = 0.297		p = 0.454	
Broad exposure in 2nd trimester		-0.411		0.002		-0.093		-0.311
		p = 0.691		p = 0.990		p = 0.483		p = 0.131
Broad exposure in 3rd trimester		-0.840		0.118		-0.086		-0.124
		p = 0.414		p = 0.477		p = 0.516		p = 0.544
Observations	636	636	636	636	636	636	636	636
Log Likelihood	-2,809.184	-2,809.743	-1,647.770	-1,648.575	-1,503.413	-1,502.503	-1,783.744	-1,783.191
Akaike Inf. Crit.	5,640.368	5,641.485	3,317.539	3,319.149	3,028.826	3,027.006	3,589.488	3,588.381

Note: *p<0.05;* ***p<0.0125;*** p<0.001

Table S5. Interactions between bushfire exposure and prior depression in predicting mental health outcomes.

	Dependent variable:							
	WHO-5		Depression		Anxiety		Stress	
Intercept	62.393***	62.361***	4.491***	4.427***	5.561***	5.428***	6.252***	6.127***
	p = 0.000	p = 0.000	p = 0.00000	p = 0.00000	p = 0.000	p = 0.000	p = 0.00000	p = 0.00000
Age, yrs	-0.131	-0.136	-0.073**	-0.071**	-0.127***	-0.124***	-0.079*	-0.075*
	p = 0.421	p = 0.404	p = 0.006	p = 0.007	p = 0.000	p = 0.000	p = 0.015	p = 0.020
Parity, number	-1.557	-1.495	0.421**	0.407**	0.516***	0.498***	0.589***	0.565***
	p = 0.063	p = 0.076	p = 0.002	p = 0.003	p = 0.00001	p = 0.00001	p = 0.0005	p = 0.001
Tertiary education (yes)	0.384	0.273	-0.439	-0.415	-0.736***	-0.703**	-0.541	-0.501
	p = 0.814	p = 0.867	p = 0.095	p = 0.115	p = 0.001	p = 0.002	p = 0.095	p = 0.122
Prior depression (yes)	-10.481***	-10.219***	1.681***	1.793***	0.918*	0.935**	1.509**	1.704**
	p = 0.0003	p = 0.0002	p = 0.0003	p = 0.00005	p = 0.016	p = 0.009	p = 0.009	p = 0.002
Prior depression (unknown)	5.628	6.229	-1.773	-2.188	-0.353	-0.615	-1.665	-2.230
	p = 0.480	p = 0.435	p = 0.168	p = 0.089	p = 0.737	p = 0.558	p = 0.293	p = 0.159
Bushfire exposure (acute)	-1.809**		0.336**		0.148		0.471***	
	p = 0.009		p = 0.003		p = 0.104		p = 0.001	
Bushfire exposure (broad)		-0.858*		0.174**		0.100*		0.256***
		p = 0.013		p = 0.002		p = 0.028		p = 0.0002
Smoke exposure (mod)	-3.129	-3.103	-0.070	-0.064	0.539	0.525	0.977	0.977
	p = 0.263	p = 0.267	p = 0.876	p = 0.887	p = 0.145	p = 0.154	p = 0.079	p = 0.078
Smoke exposure (severe)	-4.053	-3.948	0.046	0.030	0.608	0.570	1.081*	1.043*
	p = 0.129	p = 0.140	p = 0.915	p = 0.944	p = 0.085	p = 0.106	p = 0.042	p = 0.050
Acute exposure with prior depression	-0.854		0.522		0.499		0.864*	
	p = 0.660		p = 0.096		p = 0.053		p = 0.026	
Acute exposure with unknown dep history	-0.203		0.439		0.297		0.434	
	p = 0.950		p = 0.399		p = 0.485		p = 0.498	
Broad exposure with prior depression		-0.559		0.220		0.250*		0.360*
		p = 0.536		p = 0.131		p = 0.037		p = 0.045
Broad exposure with unknown dep history		-0.418		0.392		0.248		0.438
		p = 0.818		p = 0.182		p = 0.301		p = 0.225
Observations	919	919	919	919	919	919	919	919
Log Likelihood	-4,039.552	-4,039.575	-2,363.318	-2,362.340	-2,179.552	-2,176.758	-2,555.252	-2,553.510
Akaike Inf. Crit.	8,101.104	8,101.151	4,748.636	4,746.680	4,381.104	4,375.517	5,132.505	5,129.020

Note: *p<0.05;* ***p<0.0125;*** p<0.001

Table S6. Interactions between bushfire exposure and prior anxiety in predicting mental health outcomes.

	Dependent variable:			
	WHO-5	Depression	Anxiety	Stress

Intercept	65.571***	65.669***	4.550***	4.459***	5.152***	5.043***	5.502***	5.407***
	p = 0.000	p = 0.000	p = 0.00000	p = 0.00000	p = 0.000	p = 0.000	p = 0.00000	p = 0.00000
Age, yrs	-0.191	-0.189	-0.070**	-0.069**	-0.121***	-0.118***	-0.066*	-0.063*
	p = 0.230	p = 0.234	p = 0.008	p = 0.010	p = 0.00000	p = 0.00000	p = 0.038	p = 0.047
Parity, number	-1.520	-1.511	0.399**	0.393**	0.519***	0.496***	0.582***	0.554***
	p = 0.065	p = 0.067	p = 0.004	p = 0.005	p = 0.00001	p = 0.00001	p = 0.0004	p = 0.001
Tertiary education (yes)	-0.475	-0.515	-0.399	-0.380	-0.661**	-0.621**	-0.382	-0.333
	p = 0.766	p = 0.748	p = 0.133	p = 0.154	p = 0.002	p = 0.004	p = 0.228	p = 0.294
Prior anxiety (yes)	-12.935***	-13.964***	1.138***	1.335***	1.636***	1.558***	2.662***	2.679***
	p = 0.000	p = 0.000	p = 0.001	p = 0.0001	p = 0.000	p = 0.000	p = 0.000	p = 0.000
Prior anxiety (unknown)	2.983	3.213	-1.678	-2.045	0.015	-0.255	-1.046	-1.611
	p = 0.702	p = 0.680	p = 0.195	p = 0.115	p = 0.989	p = 0.806	p = 0.499	p = 0.297
Bushfire exposure (acute)	-1.982**		0.273*		0.225*		0.550***	
	p = 0.008		p = 0.028		p = 0.024		p = 0.0003	
Bushfire exposure (broad)		-1.119**		0.160**		0.132**		0.292***
		p = 0.004		p = 0.011		p = 0.009		p = 0.0001
Smoke exposure (moderate)	-0.746	-0.737	-0.410	-0.410	0.265	0.248	0.500	0.487
	p = 0.785	p = 0.787	p = 0.366	p = 0.366	p = 0.466	p = 0.493	p = 0.356	p = 0.368
Smoke exposure (severe)	-1.928	-1.798	-0.222	-0.246	0.369	0.325	0.663	0.609
	p = 0.460	p = 0.491	p = 0.608	p = 0.572	p = 0.289	p = 0.348	p = 0.201	p = 0.239
Acute exposure with prior anxiety	-0.130		0.475*		-0.022		0.156	
	p = 0.925		p = 0.036		p = 0.902		p = 0.565	
Acute exposure with unknown anxiety history	0.040		0.504		0.214		0.340	
	p = 0.990		p = 0.338		p = 0.612		p = 0.589	
Broad exposure with prior anxiety		0.395		0.157		0.027		0.076
		p = 0.543		p = 0.146		p = 0.753		p = 0.553
Broad exposure with unknown anxiety history		-0.110		0.404		0.211		0.391
		p = 0.951		p = 0.174		p = 0.373		p = 0.269
Observations	919	919	919	919	919	919	919	919
Log Likelihood	-4,017.434	-4,017.313	-2,368.583	-2,368.501	-2,164.805	-2,162.017	-2,531.876	-2,529.335
Akaike Inf. Crit.	8,056.867	8,056.626	4,759.166	4,759.003	4,351.610	4,346.033	5,085.752	5,080.670
Note:								*p<0.05;* ***p<0.0125;*** p<0.001

Table S7. Interactions between bushfire exposure and living with spouse in predicting mental health outcomes.

	Dependent variable:							
	WHO-5		Depression		Anxiety		Stress	
Intercept	56.597***	55.677***	6.406***	6.388***	7.323***	7.422***	8.235***	8.284***
	p = 0.000	p = 0.000	p = 0.00000	p = 0.00000	p = 0.000	p = 0.000	p = 0.00000	p = 0.00000

Age, yrs	-0.040	-0.047	-0.089**	-0.087**	-0.141***	-0.138***	-0.098**	-0.095**
	p = 0.810	p = 0.779	p = 0.002	p = 0.002	p = 0.000	p = 0.000	p = 0.004	p = 0.005
Parity, number	-1.577	-1.540	0.445**	0.429**	0.545***	0.532***	0.617***	0.593***
	p = 0.067	p = 0.074	p = 0.002	p = 0.003	p = 0.00001	p = 0.00001	p = 0.0004	p = 0.001
Tertiary education (yes)	1.581	1.493	-0.689**	-0.651*	-0.851***	-0.818***	-0.811*	-0.759*
	p = 0.342	p = 0.371	p = 0.012	p = 0.017	p = 0.0002	p = 0.0003	p = 0.016	p = 0.023
Living with spouse (yes)	-0.195	0.866	-0.919	-0.965	-1.063	-1.306*	-0.864	-1.044
	p = 0.971	p = 0.862	p = 0.289	p = 0.234	p = 0.129	p = 0.046	p = 0.416	p = 0.293
Bushfire exposure (acute)	-2.307		-0.054		0.123		0.340	
	p = 0.415		p = 0.908		p = 0.741		p = 0.549	
Bushfire exposure (broad)		-0.763		-0.032		0.006		0.126
		p = 0.533		p = 0.874		p = 0.970		p = 0.607
Smoke exposure (moderate)	-2.509	-2.494	-0.166	-0.175	0.462	0.446	0.881	0.863
	p = 0.379	p = 0.382	p = 0.722	p = 0.708	p = 0.219	p = 0.234	p = 0.123	p = 0.130
Smoke exposure (severe)	-3.916	-3.809	0.043	0.0001	0.577	0.529	1.083*	1.017
	p = 0.151	p = 0.163	p = 0.924	p = 1.000	p = 0.108	p = 0.141	p = 0.048	p = 0.063
Acute exposure while living with spouse	0.524		0.467		0.083		0.231	
	p = 0.857		p = 0.325		p = 0.828		p = 0.690	
Broad exposure while living with spouse		-0.140		0.254		0.134		0.191
		p = 0.912		p = 0.219		p = 0.420		p = 0.451
Observations	919	919	919	919	919	919	919	919
Log Likelihood	-4,061.932	-4,062.054	-2,397.974	-2,397.066	-2,200.174	-2,197.979	-2,584.173	-2,582.389
Akaike Inf. Crit.	8,141.864	8,142.107	4,813.948	4,812.132	4,418.348	4,413.957	5,186.346	5,182.778

Note: *p<0.05;* ***p<0.0125;*** p<0.001

Table S8. Interactions between bushfire exposure and household income in predicting mental health outcomes.

	Dependent variable:							
	WHO-5		Depression		Anxiety		Stress	
Intercept	55.679***	57.613***	5.517***	5.579***	7.085***	7.059***	7.470***	7.395***
	p = 0.000	p = 0.000	p = 0.00001	p = 0.00001	p = 0.000	p = 0.000	p = 0.00000	p = 0.00000
Age, yrs	-0.048	-0.047	-0.089**	-0.087**	-0.133***	-0.130***	-0.101**	-0.098**
	p = 0.776	p = 0.781	p = 0.002	p = 0.002	p = 0.000	p = 0.000	p = 0.003	p = 0.004
Parity, number	-1.575	-1.521	0.428**	0.413**	0.522***	0.503***	0.625***	0.593***
	p = 0.067	p = 0.077	p = 0.003	p = 0.004	p = 0.00001	p = 0.00001	p = 0.0003	p = 0.001
Tertiary education (yes)	1.577	1.499	-0.692**	-0.665*	-0.855***	-0.824***	-0.908**	-0.858**
	p = 0.346	p = 0.371	p = 0.012	p = 0.016	p = 0.0002	p = 0.0003	p = 0.007	p = 0.011
Income (high)	1.020	-1.177	0.018	-0.095	-1.068	-1.164	0.086	0.046
	p = 0.867	p = 0.842	p = 0.986	p = 0.922	p = 0.183	p = 0.135	p = 0.944	p = 0.969
Bushfire exposure (acute)	-1.584		0.444		0.003		0.039	
	p = 0.576		p = 0.339		p = 0.994		p = 0.945	
Bushfire exposure (broad)		-1.330		0.187		0.001		0.032
		p = 0.294		p = 0.367		p = 0.996		p = 0.899
Smoke exposure (moderate)	-2.513	-2.481	-0.168	-0.179	0.465	0.451	0.891	0.879
	p = 0.378	p = 0.385	p = 0.720	p = 0.702	p = 0.217	p = 0.231	p = 0.119	p = 0.123
Smoke exposure (severe)	-3.935	-3.811	0.047	0.012	0.604	0.561	1.093*	1.033
	p = 0.149	p = 0.163	p = 0.916	p = 0.980	p = 0.094	p = 0.119	p = 0.045	p = 0.058
Acute exposure with high income	-0.237		-0.054		0.213		0.563	
	p = 0.935		p = 0.910		p = 0.578		p = 0.332	
Broad exposure with high income		0.465		0.020		0.141		0.300
		p = 0.722		p = 0.925		p = 0.413		p = 0.250
Observations	919	919	919	919	919	919	919	919
Log Likelihood	-4,061.945	-4,061.998	-2,398.561	-2,397.910	-2,201.347	-2,199.255	-2,583.164	-2,581.185
Akaike Inf. Crit.	8,141.890	8,141.996	4,815.122	4,813.819	4,420.693	4,416.511	5,184.327	5,180.369
Note:								*p<0.05;* **p<0.0125;** p<0.001

Table S9. Interactions between bushfire exposure and parity in predicting mental health outcomes.

	Dependent variable:							
	WHO-5		Depression		Anxiety		Stress	
Intercept	55.987***	56.025***	5.551***	5.576***	6.493***	6.360***	7.522***	7.472***
	p = 0.000	p = 0.000	p = 0.000	p = 0.000	p = 0.000	p = 0.000	p = 0.000	p = 0.000
Age, yrs	-0.040	-0.045	-0.089***	-0.088**	-0.139***	-0.136***	-0.098**	-0.095**
	p = 0.808	p = 0.784	p = 0.001	p = 0.002	p = 0.000	p = 0.000	p = 0.004	p = 0.005
Parity, number	-1.074	-0.974	0.423*	0.327	0.277	0.247	0.469	0.367
	p = 0.402	p = 0.439	p = 0.044	p = 0.113	p = 0.102	p = 0.136	p = 0.069	p = 0.145
Tertiary education (yes)	1.567	1.454	-0.696**	-0.657*	-0.878***	-0.840***	-0.824*	-0.764*
	p = 0.344	p = 0.381	p = 0.011	p = 0.016	p = 0.0001	p = 0.0002	p = 0.013	p = 0.022
Bushfire exposure (acute)	-1.465		0.388**		0.038		0.472**	
	p = 0.113		p = 0.011		p = 0.758		p = 0.011	
Bushfire exposure (broad)		-0.696		0.175*		0.040		0.230*
		p = 0.136		p = 0.023		p = 0.511		p = 0.014
Smoke exposure (moderate)	-2.471	-2.465	-0.168	-0.184	0.448	0.438	0.872	0.852
	p = 0.386	p = 0.387	p = 0.719	p = 0.694	p = 0.234	p = 0.243	p = 0.127	p = 0.135
Smoke exposure (severe)	-3.885	-3.816	0.047	0.010	0.577	0.549	1.083*	1.030
	p = 0.154	p = 0.162	p = 0.917	p = 0.983	p = 0.109	p = 0.127	p = 0.048	p = 0.059
Acute exposure with additional child	-0.380		0.005		0.190*		0.101	
	p = 0.595		p = 0.967		p = 0.045		p = 0.479	
Broad exposure with additional child		-0.211		0.033		0.102*		0.080
		p = 0.549		p = 0.565		p = 0.029		p = 0.254
Observations	919	919	919	919	919	919	919	919
Log Likelihood	-4,061.819	-4,061.887	-2,398.574	-2,397.748	-2,200.532	-2,198.118	-2,584.329	-2,582.291
Akaike Inf. Crit.	8,139.639	8,139.775	4,813.147	4,811.495	4,417.064	4,412.236	5,184.657	5,180.582

Note: *p<0.05;* ***p<0.0125;*** p<0.001

Table S10. Interactions between bushfire exposure and setting (urban/rural) in predicting mental health outcomes.

	Dependent variable:							
	WHO-5		Depression		Anxiety		Stress	
Intercept	56.636***	56.557***	5.582***	5.497***	6.196***	5.981***	7.414***	7.241***
	p = 0.000	p = 0.000	p = 0.000	p = 0.000	p = 0.000	p = 0.000	p = 0.000	p = 0.000
Age, yrs	-0.051	-0.058	-0.088**	-0.086**	-0.137***	-0.133***	-0.095**	-0.091**
	p = 0.757	p = 0.725	p = 0.002	p = 0.002	p = 0.000	p = 0.000	p = 0.005	p = 0.006
Parity, number	-1.699*	-1.620	0.442**	0.423**	0.539***	0.515***	0.631***	0.598***
	p = 0.047	p = 0.059	p = 0.002	p = 0.003	p = 0.00001	p = 0.00001	p = 0.0003	p = 0.0005
Tertiary education (yes)	1.813	1.698	-0.721**	-0.686**	-0.916***	-0.887***	-0.888**	-0.832**
	p = 0.272	p = 0.305	p = 0.008	p = 0.012	p = 0.00004	p = 0.0001	p = 0.008	p = 0.012
Setting (rural)	4.110	4.875	-0.752	-0.516	-0.067	0.182	-1.343	-1.038
	p = 0.286	p = 0.188	p = 0.234	p = 0.395	p = 0.895	p = 0.710	p = 0.082	p = 0.161
Bushfire exposure (acute)	-2.622**		0.426**		0.300**		0.697***	
	p = 0.002		p = 0.002		p = 0.006		p = 0.00002	
Bushfire exposure (broad)		-1.223**		0.239***		0.211***		0.397***
		p = 0.003		p = 0.0004		p = 0.0001		p = 0.00001
Smoke exposure (moderate)	-2.076	-2.049	-0.218	-0.226	0.446	0.432	0.775	0.759
	p = 0.467	p = 0.473	p = 0.641	p = 0.630	p = 0.238	p = 0.251	p = 0.175	p = 0.183
Smoke exposure (severe)	-3.528	-3.399	0.003	-0.033	0.574	0.528	0.997	0.932
	p = 0.195	p = 0.213	p = 0.996	p = 0.941	p = 0.112	p = 0.142	p = 0.068	p = 0.087
Acute exposure in rural setting	0.803		0.093		-0.169		0.014	
	p = 0.596		p = 0.710		p = 0.401		p = 0.965	
Broad exposure in rural setting		0.171		-0.015		-0.171		-0.080
		p = 0.817		p = 0.903		p = 0.078		p = 0.587
Observations	919	919	919	919	919	919	919	919
Log Likelihood	-4,059.041	-4,059.400	-2,397.469	-2,396.852	-2,201.432	-2,197.858	-2,580.879	-2,578.897
Akaike Inf. Crit.	8,136.081	8,136.801	4,812.938	4,811.705	4,420.864	4,413.715	5,179.759	5,175.795
Note:						*p<0.05;*	***p<0.0125;***	p<0.001
